# Radiation dose optimisation in biomedical imaging and intervention

**DOI:** 10.2349/biij.3.2.e50

**Published:** 2007-04-01

**Authors:** MM Rehani

**Affiliations:** International Atomic Energy Agency, Vienna, Austria

Situations in life are never simply black and white. There are many shades of gray. The gray zones provide a lot of playing ground for scientists engaged in optimisation. We all understand what is meant by optimisation, such as “the procedure or procedures used to make a system or design as effective or as functional as possible” or “making the best of anything”. Thus, the driving force is getting to the “best”. For example, in medical imaging, one attempts to choose certain parameters to optimise, such as image quality and patient dose. Both do not go in the same direction. If one increases image quality (which is desirable), one ends up increasing patient dose too (which is not desirable). For radiologists, image quality is the main focus. How often does one hear a radiologist asking, while reporting an imaging investigation, “how much radiation dose has been imparted to the patient”? Similarly, physicists are concerned with radiation dose. It is again not common to see a physicist asking himself what image quality is associated with the dose he estimated in an imaging procedure. Optimisation, therefore, tends to imply the best image quality for a radiologist and the least radiation dose for a physicist. Neither is desirable without regard for the other. Thus, the dictionary meaning of “optimisation”, in particular “making the best of anything” is not meaningful in isolation. The best may be happening in both but what we want to achieve works in opposite directions for the two parameters. To make things simpler, it is somewhat similar to the quality of consumer goods that we buy and the price that we have to pay. Higher quality involves higher price but we want to create a balance between quality and affordable cost.

In this special thematic issue of Biomedical Imaging and Intervention Journal (BIIJ), 12 papers contributed by authors from 11 countries have reviewed the situation in conventional and digital radiography, fluoroscopy, mammography, PET/CT, computed tomography and radiotherapy. Educational issues for cardiologist on radiation protection and accident prevention in radiotherapy, both of which have important role in optimisation, have also been covered.

A good quality assurance (QA) program should reduce the need for monitoring. This has been documented by Vano and Fernandez in their paper on dose management in digital radiography. They present the experience on an online audit tool for digital radiography that they had developed. Working on images obtained on many thousands of patients for mammography, chest radiography, computed radiography and interventional radiology procedures, they show that very few alarm signals were generated. While continuous monitoring by people is an undesirable tool in quality management, an automatic online audit system is certainly helpful.

Optimisation in general radiography still poses a big challenge. The variation in doses in large scale surveys are quite high and in this respect a paper by Colin Martin reviews the methods that can be used for optimisation. Understanding the role of parameters like kV, filtration, screen-film combination, anti-scatter grid and automatic exposure control is crucial. Diagnostic reference levels have proved their value in optimisation and this has been emphasized.

Computed tomography (CT) has continued to pose challenge in optimisation. At the rate technology has been progressing during the past 7 years, it has overtaken effective optimisation actions in practice. Virginia Tsapaki and Madan Rehani in their review cover some aspects of optimisation in CT indicating the important role users need to play in day-to-day management of situations in clinical practice.

In view of lack of practical experience in radiation-induced injuries among medical professionals, diagnosis of such injuries has often followed a tortuous path. Louis Wagner reviews the characteristics of radiation-induced injuries to the skin and some actions that can be taken to reduce their likelihood or severity. Failure of optimisation can result in injuries. This awareness is important and in that respect an article by Madan Rehani in this issue presents the role of International Atomic Energy Agency (IAEA) on interventional cardiologists training in radiation protection. With interventional cardiologists being intensive user of radiation and having minimal or no training, this issue attains great importance.

Optimisation in radiotherapy has been dominated by precision in dose delivery to target tissue as primary goal and reduction of dose to surrounding normal tissue as a secondary goal. Paul Ravindran in his review covers optimisation while using imaging tools such as electronic portal imaging and cone beam CT, which are used prior to delivery of radiation so as to visualize the organ to be treated. It is now being recognized that repeated use of the imaging system for 25 to 30 fractions could give considerable dose to normal tissue and critical organs.

Ola Holmberg in his paper “Accident prevention in radiotherapy” describes lessons learned from major radiotherapy accidents in order to highlight patterns seen where accidents have occurred and identify preventive actions. Although optimisation itself is a tool for accident prevention, however, the reverse is also true. The lessons from earlier accidents provide basis for optimisation.

Physicians generally administer similar levels of activity or activity per unit total body mass to all patients. This has been reasonably successful in the use of radioiodine against thyroid cancer and hyperthyroidism wherein the “therapeutic window” (difference in dose levels between what is experienced by the tumour and that experienced by the most important normal tissue) is large. Mike Stabin and Glenn Flux discuss the need for patient-individualised dose calculations to optimise therapy for patients, provide improved clinical outcome and minimise the risk of unwanted side effects.

Optimisation in the decade-old technology of PET/CT has many issues pertaining to scanning protocols and artefacts produced by motion. Habib Zaidi reviews the situation and indicates that differences between PET and CT breathing protocols might lead to misalignment artefacts owing to anatomical dislocations of the diaphragm and chest wall during a PET/CT scan. This requires caution while interpreting a study where patient is suffering from disease in periphery of the lung. Attention has been drawn to many technological barriers still existing.

Bertil Axelsson makes a case of comprehensive quality systems in fluoroscopy to optimise the patient dose and image quality.

